# Determination of Susceptibility Breakpoint for Cefquinome against *Streptococcus suis* in Pigs

**DOI:** 10.3390/antibiotics10080958

**Published:** 2021-08-09

**Authors:** Kun Mi, Mei Li, Lei Sun, Yixuan Hou, Kaixiang Zhou, Haihong Hao, Yuanhu Pan, Zhenli Liu, Changqing Xie, Lingli Huang

**Affiliations:** 1 National Reference Laboratory of Veterinary Drug Residues (HZAU) and MAO Key Laboratory for Detection of Veterinary Drug Residues, Huazhong Agricultural University, Wuhan 430070, China; mikun@webmail.hzau.edu.cn (K.M.); mli9393@163.com (M.L.); sunlei23@webmail.hzau.edu.cn (L.S.); haohaihong@mail.hzau.edu.cn (H.H.); liuzhli009@mail.hzau.edu.cn (Z.L.); xiechangqing@mail.hzau.edu.cn (C.X.); 2 MOA Laboratory for Risk Assessment of Quality and Safety of Livestock and Poultry Products, Huazhong Agricultural University, Wuhan 430070, China; hyx97@webmail.hzau.edu.cn (Y.H.); flyingkai@webmai.hzau.edu.cn (K.Z.); panyuanhu@mail.hzau.edu.cn (Y.P.)

**Keywords:** cefquinome, *Streptococcus suis*, clinical breakpoint, epidemiological cutoff, PD cutoff, clinical cutoff, dosage regimen, PK/PD model

## Abstract

*Streptococcus suis* (*S. suis*), a zoonotic pathogen, causes severe diseases in both pigs and human beings. Cefquinome can display excellent antibacterial activity against gram-negative and gram-positive bacteria. The aim of this study was to derive an optimal dosage of cefquinome against *S. suis* with a pharmacokinetic/pharmacodynamic (PK/PD) integration model in the target infection site and to investigate the cutoffs monitoring the changes of resistance. The minimum inhibitory concentration (MIC) distribution of cefquinome against 342 *S. suis* strains was determined. MIC_50_ and MIC_90_ were 0.06 and 0.25 μg/mL, respectively. The wild-type cutoff was calculated as 1 μg/mL. A two-compartmental model was applied to calculate the main pharmacokinetic parameters after 2 mg/kg cefquinome administered intramuscularly. An optimized dosage regimen of 3.08 mg/kg for 2-log_10_ CFU reduction was proposed by ex vivo PK/PD model of infected swine. The pharmacokinetic-pharmacodynamic cutoff was calculated as 0.06 μg/mL based on PK/PD targets. Based on the clinical effectiveness study of pathogenic MIC isolates, the clinical cutoff was calculated as 0.5 μg/mL. A clinical breakpoint was proposed as 1 μg/mL. In conclusion, the results offer a reference for determining susceptibility breakpoint of cefquinome against *S. suis* and avoiding resistance emergence by following the optimal dosage regimen.

## 1. Introduction

*Streptococcus suis* (*S. suis*) is a gram-positive facultative anaerobe and increasingly emerging zoonotic infection with a global distribution [1]. Among the 35 serotypes (1/2, 1–34) of *S. suis* classified by different capsule antigens, pathogenicity has been reported for serotype 7 strains isolated from diseased animals and humans [2]. Increasing emergence and dissemination of resistance has been reported in recent years, mainly caused by exposure to antibacterial abuse and misuse.

**Figure 1:**
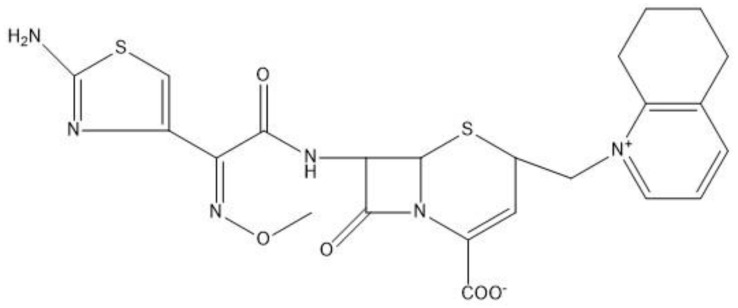
Chemical structure of cefquinome.

Cefquinome is a fourth-generation cephalosporin developed exclusively for veterinary use in the clinic [3]. Due to its structure characteristic (Figure 1), cefquinome displays outstanding stability for beta-lactamase and exerts excellent antibacterial activity against gram-positive and gram-negative bacteria in vitro or in vivo antibacterial activity [4]. It has been approved for the treatment of respiratory tract infections with a dosage of 2 mg/kg by the European Medicines Agency (EMA) [5].

The susceptibility breakpoint (SBPT) is useful to determine the susceptibility or resistance of a bacterium to an antibiotic and to define the effect, or inappropriate antibiotic treatments, on bacterial infections. The setting breakpoint is composed of three parts: in vitro susceptibility, in vivo pharmacokinetics of animals, and clinical or bacteriological outcome [6]. Three terms, epidemiological cutoff (ECOFF), pharmacodynamic cutoff (CO_PD_), and clinical cutoff (CO_CL_) are employed to describe these concepts [7,8]. ECOFF is a useful tool for laboratories conducting susceptibility testing and for clinicians testing infections. CO_PD_ is the highest MIC which can achieve probable target attainment (PTA) (>90%) in the population from the target PK/PD index by Monte Carlo simulation. The CO_CL_ is a MIC-related clinical outcome. There is no previous investigation studying the susceptibility breakpoints which contains the establishment of a clinical cutoff for cefquinome against *S. suis*. Irrational use of antibiotics in the veterinary clinic is the major reason leading to resistance. The pharmacokinetic/pharmacodynamic (PK/PD) model can design an optimal dosing regimen to avoid resistance development [9]. To our knowledge, no PK/PD model of cefquinome against *S. suis* has been developed in swine. It is useful to assess the dose of cefquinome against *S. suis*, in order to guide clinical therapy.

In this study, a terminal ECOFF (TECOFF), CO_PD_, and CO_CL_ of cefquinome against *S. suis* are explored which can provide evidence for the susceptibility breakpoint. The dosage regimens of cefquinome against *S. suis* are optimized by an ex vivo PK/PD model. This will be influential in protecting the efficacy of cefquinome.

## 2. Results

### 2.1. MIC Distribution of CEF against S. suis

**Figure 2:**
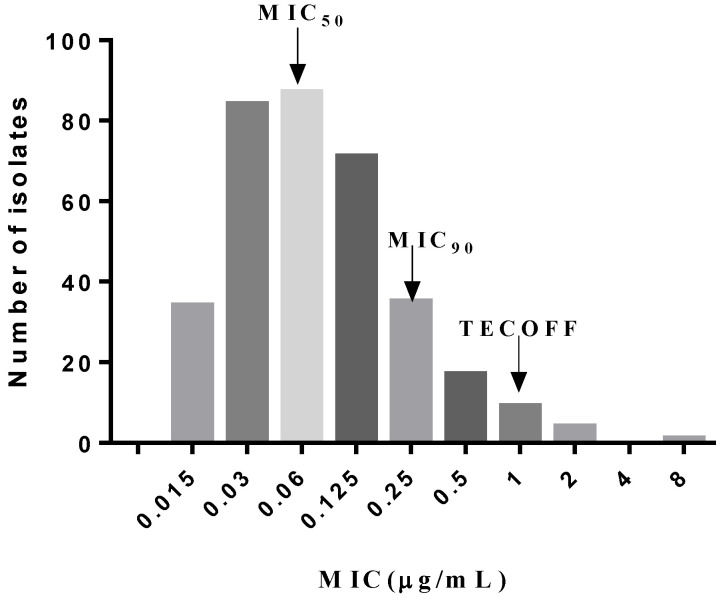
MIC distribution of cefquinome against 342 *S. suis* isolates.

The distribution of cefquinome against 342 *S. suis* isolates is shown in Figure 2. MIC_50_ and MIC_90_ for cefquinome against *S. suis* were determined to be 0.06 and 0.25 μg/mL, respectively. The minimum inhibitory concentration (MIC) data of 342 *S. suis* isolates showed that the MIC distribution was consistent with a normal distribution by normality test (*p* > 0.2). The upper tail (99.9%) of the wild-type cutoff value was 1 μg/mL. The optimum MIC range (0.015–1 μg/mL) was obtained using the NORMINV function, and the probability of an isolate with an MIC value higher than the high cutoff was lower than 1.5% when employing the NORMDIST function. The difference between the estimate (340) and the actual isolate number (337) was minimal when the optimum fit was 1 μg/mL, and 98.5% of the wild-type isolates were included in the ECOFF. As a result, the tentative ECOFF was defined as 1 μg/mL (Table 1). The ECOFF of cefquinome against *S. suis* needs more results from different laboratories to be ultimately determined [10].

### 2.2. MIC, MBC, MPC and PAE of Cefquinome against SS0061

The MIC values of cefquinome against *SS0061* were both 0.125 μg/mL in vitro and ex vivo, and the minimum bactericidal concentration (MBC) values were 0.125 and 0.25 μg/mL in vitro and ex vivo, respectively. The mutant prevention concentration (MPC) value of cefquinome against *SS0061* was 0.4 μg/mL, and the mutant selection window (MSW) was 0.125–0.4 μg/mL, indicating that the resistant mutant selection window was narrow. Post-antibiotic effect (PAE) of different concentrations of cefquinome (1×, 2×, or 4× MIC) and exposure time (1 or 2 h) are shown in Table 2. With an increase in drug concentrations and exposure time, the PAEs are enhanced.

### 2.3. In Vitro and Ex Vivo Time-Killing Curves

**Figure 3:**
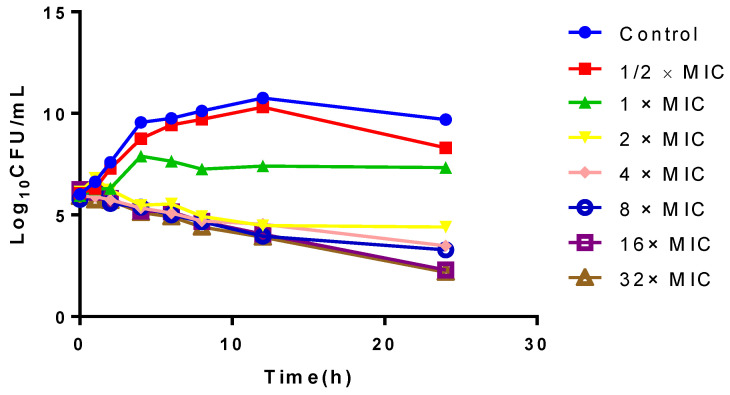
The in vitro time-killing curves of cefquinome against *SS0061*.

The in vitro time-killing curves of cefquinome against *SS0061* are illustrated in Figure 3. The drug can inhibit the growth of *S. suis* after exposure 1 × MIC for 24 h, along with a visible growth inhibition, whereas >2 log_10_ CFU/mL reductions in bacterial density occurred after exposure to cefquinome at 4 × MIC for 24 h. After 24 h incubations with high drug concentrations (16 × MIC~32 × MIC), *S. suis* can maintain a bacterial density of about 2 log_10_ CFU/mL.

**Figure 4:**
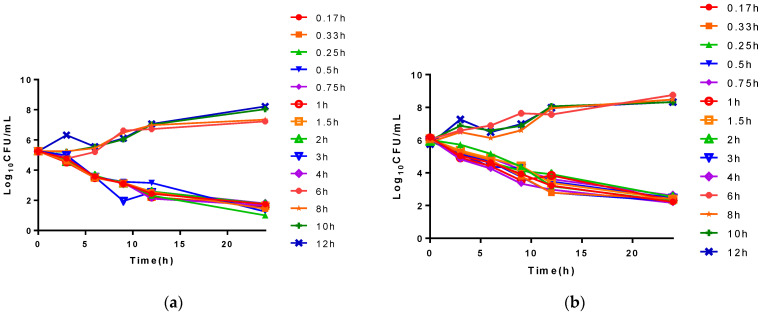
The ex vivo time-killing curves of cefquinome against *S. suis.* (**a**) represents in healthy group plasma; (**b**)represents in infected group plasma. The legends mean different plasma samples after cefquinome administration.

The ex vivo time-killing curves are shown in Figure 4. The bacteria were reduced drastically to about 3 log_10_ CFU/mL exposure to the plasma samples after intramuscular (I.M) administration.

### 2.4. Pharmacokinetic Analysis of Cefquinome in Plasma

**Figure 5:**
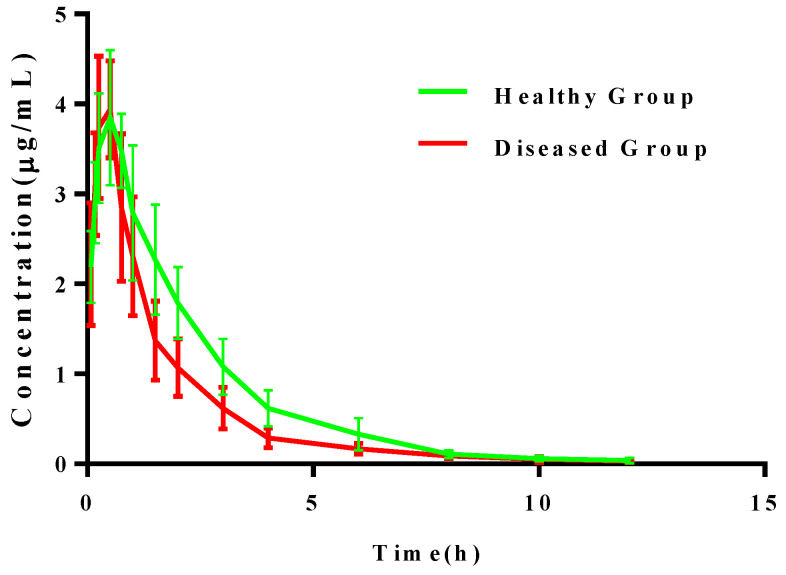
Concentration-time of cefquinome in plasma by 2 mg/kg b.w. intramuscular injection.

The concentration-time profiles of cefquinome in plasma were described by a two-compartmental open model with a first-order absorption phase after a single I.M. administration in healthy and infected pigs. The main PK parameters of cefquinome are illustrated in Table 3. The concentration-time profiles are presented in Figure 5.

The AUC of plasma in both healthy pigs (9.77 ± 0.63 h × μg/mL) and infected pigs (9.86 ± 0.78 h × μg/mL) was similar. C_max_ values of 3.93 ± 0.11 μg/mL (healthy) and 3.92 ± 0.13 (*S. suis*-infected) were reached at 0.45 ± 0.02 h and 0.42 ± 0.03 h, respectively, indicating that the concentration of cefquinome in the plasma of the healthy and infected groups was similar. CL/F is also similar in healthy and infected groups indicating that the clearance rate is not influenced by healthy conditions.

### 2.5. PK/PD Integration Modeling and Dose Estimation

The PK/PD profiles of concentrations versus antibacterial effects in plasma were simulated by the inhibitory sigmoid *I_max_* model. The correlation between the antimicrobial effect and ex vivo PK/PD parameters of (AUC_24 h_/MIC)_ex_ was simulated and the parameters are illustrated in Table 4. Ultimately, the corresponding dose of cefquinome achieving bacteriostasis is 2.86 mg/kg, 1-log_10_ killing effect is 2.96 mg/kg and 2-log_10_ killing is 3.08 mg/kg.

### 2.6. Determination of CO_PD_

**Figure 6:**
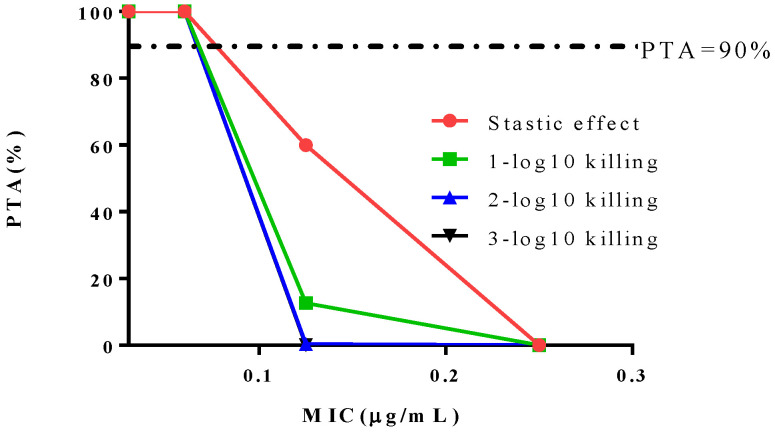
Probability of target attainment (PTA) for different antibacterial effects on the healthy group.

PK/PD indices (AUC_24 h_/MIC) for different antibacterial effect were selected as the target value. 10,000 subjects were simulated by Monte Carlo simulation. Ultimately, the CO_PD_ for cefquinome against *S. suis* was defined as 0.06 μg/mL (as Figure 6 shows).

### 2.7. Clinical Outcomes and Determination of CO_CL_

When the experimental model of pig streptococcosis was successfully established, the pigs exhibited obvious clinical symptoms, such as roughened body coats, loss of appetite, and elevated body temperature (39.5 to 42.0 °C); the pigs were reluctant to rise, and some were lame in one or more legs. A few of the pigs exhibited severe central nervous system signs, such as head tilt, tremors, prostration, and opisthotonos.

The clinical outcomes corresponded to pig *streptococcosis* caused by 5 isolates of *S. suis* and treatment with a recommended dosage of cefquinome (2 mg/kg). The results of clinical experiment are shown in Table 5. The clinical cure rate (no relapse over 7 days following cure) was 100% using a therapeutic dosing regimen in the *SSZD01* and *SS1496* groups. The cure rates were 83%, 67%, and 50% in the *SS0061*, *SS14130*, and *SS2481* groups, respectively.

The clinical outcomes were analyzed by the WindoW approach [11]. The calculation and clinical experiment results are shown in Table 5. The cure rate and mortality rate of 5 isolates with different MICs in the treatment group were statistically analyzed, and the survival animals of the treatment group were 6, 6, 5, 4, and 3. The probabilities calculated by CAR algorithms were 100, 100, 83.33, 66.67, and 50. The MIC of the isolate corresponding to the minimum value of CAR was selected as 1 μg/mL. Meanwhile, the CAR should not be set at the lowest or highest measured MIC. The difference in the cure probability of each MIC calculated by the MaxDiff algorithm was 25.00, 33.30, 25.03, 16.67, and 0. The MIC of the isolate corresponding to the maximum MaxDiff value was selected as the lower limit of the cutoff at 0.06 μg/mL. As a result, the window of CO_CL_ was 0.06–1 μg/mL.

**Figure 7:**
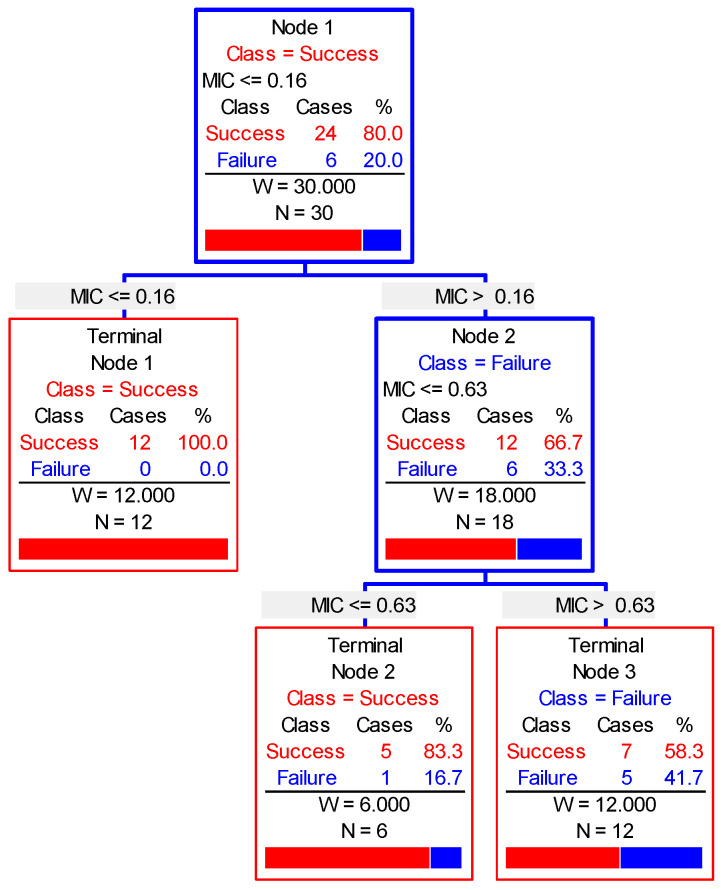
The simulation of CART modeling.

The CART algorithm was applied to analyze the clinical outcomes of each pig (Figure 7). The cure rate reached 100% when cefquinome was added at MIC values ≤0.16 μg/mL. However, the cure rate was close to 83.3% when cefquinome was added at MIC values ≤0.63 μg/mL. The CO_CL_ is the MIC at which the cure rate equals 90%. Consequently, the CO_CL_ value ranged from 0.16 to 0.63 μg/mL.

Nonlinear regression analysis was performed to obtain the equation expressing the relationship between log_2_ MIC and cure rate. The equation was y = −1.144x^2^ − 12.831x + 64.812, and R^2^ (the correlation coefficient) was 0.99. When the cure rate equaled 90%, the corresponding log_2_ MIC value was approximately −1, and the MIC value was 0.5 μg/mL, which accorded with the range of 0.16–0.63 μg/mL. Consequently, the CO_CL_ was defined as 0.5 μg/mL.

### 2.8. Development of Susceptibility Breakpoints

The results of TECOFF > CO_CL_ > CO_PD_ were obtained by comparing the TECOFF and CO_PD_, and CO_CL_ values. Ultimately, according to the susceptibility breakpoint decision tree, the final susceptibility breakpoint was defined as 1 μg/mL.

## 3. Discussion

*S. suis* is widely regarded as one of the most important zoonotic pathogens causing severe infection in animals and humans, with clinical symptoms such as arthritis, encephalitis, and pneumonia [12]. It is essential to optimize the dosage regimen and monitor the development of resistance of *S. suis*, because of the rapid spread of resistance to common antibiotics.

The ECOFF is used to distinguish the distribution of wild-type and non-wild type bacterial populations [13]. The traditional method for determining ECOFF is a visual inspection of distribution histograms of the MICs of special agents against organisms. However, this method may be improper when there is a bimodal MIC distribution, and the determination lacks reproducibility because the calculation process is observer-dependent [14]. In this study, we adapted a statistical technique process to determine the TECOFF as 1 μg/mL with nonlinear regression analysis fitting to a cumulative log-normal MIC distribution [14], and this value was similar to the result by visual inspection. The ECOFF of cefquinome against *S. suis* needs at least five MIC distributions from different laboratories. The TECOFF can provide evidence for the determination of ECOFF.

Monte Carlo simulation is a simple and semi-random number generator that merges the variable distribution of population PK/PD parameters and the PTA. This simulation can describe the relationship of random probability. As the simulation proceeds, 10,000 AUC/MIC ratios are calculated with their probabilities, and then all AUC/MIC ratio values and the probability of attaining each of them are obtained [15]. In this study, the CO_PD_ was calculated as 0.06 μg/mL. This value was equal to the MIC_50_ of cefquinome against 342 *S. suis* isolates, indicating that half of the *S. suis* infections could be cured by using cefquinome under the recommended regimen [16]. CO_PD_ of cefquinome against *haemophilus parasuis* was also calculated as 0.06 μg/mL [17].

The PK/PD model is a valuable tool to optimize dosage regimen [18]. An accurate PK/PD integration model is essential to predict the clinical treatment efficacy of antibiotics for respiratory tract infection and establish an interpretive susceptibility breakpoint for preventing the spread of bacterial resistance. %T > MIC is the most appropriate PK/PD index for a time-dependent antibiotic to determine antibacterial efficacy and predict therapeutic efficacy. However, the ex vivo antibacterial pattern of cefquinome was altered and different from the pattern of the in vitro conditions in the time-killing curve experiment. As the concentration increased in serum, the bacterial counts decreased after 24 h of incubation, indicating that the antibacterial activity is concentration-related under ex vivo conditions. In addition, the different parameters based on the values of T > MIC and bacterial counts (log_10_ CFU/mL) cannot be acquired using ex vivo PK/PD integration, and AUC/MIC is also used for describing the character of antibacterial activity of cefquinome [19]. For these reasons, AUC/MIC was regarded as the most appropriate PK/PD index to describe the antibacterial activity of cefquinome in serum.

EMA have proposed a dose of cefquinome to treat respiratory tract infections of swine as 2 mg/kg bodyweight. The recommend dose is designed for respiratory tract disease, not only for *S. suis* infection. The PK/PD model is recognized to evaluate and optimize the dose regimen and guide clinical treatment. There are many previous studies investigating the dose of cefquinome against a specific bacterium [19,20,21]. From the manuscript, the ECOFF, CO_PD_ and CO_CL_ were determined as 1 μg/mL, 0.06 μg/mL and 0.5 μg/mL, respectively. Compared with CO_PD_ and ECOFF, CO_PD_ is less than ECOFF which indicates the recommended dose regimen is a little bit too low to treat the wild-type population infected by *S. suis* [22]. From the clinical experiment, for the pathogens associated with high MIC values, the cure rate is less than 67% at a dose of 2 mg/kg, indicating the dose of cefquinome against *S. suis* needs to be optimized. By the current ex vivo PK/PD model for the infected group, the dose is determined as 3.08 mg/kg. From our viewpoint, it is important to determine the MIC of cefquinome against *S. suis* before antibacterial treatment. If the MIC is higher than the clinical breakpoint, it is a better way to select the optimized dose (3.08 mg/kg) or change to another antibiotic. When the MIC is lower than the clinical breakpoint, the recommended dose is still the best choice.

The correlation of MICs with the clinical outcome of infection caused by causative pathogens is important for establishing clinical cutoffs. The CO_CL_ was developed and proposed by inspecting clinical and microbiological outcomes based on the MICs associated with clinical infection. The decisive factors associated with establishing CO_CL_ include the MICs of the causative isolates of infection, the dosage regimen of antibiotics, the pharmacokinetics in target animals, the toxicological aspects, comprehensive microbiological studies, the clinical outcome aspects, and so on. In a previous study, the clinical breakpoints of amoxicillin to the pathogens of causative porcine respiratory tract infection were established as ≤0.5 μg/mL for “susceptible”, 1 μg/mL for “intermediate”, and ≥2 μg/mL for “resistant” based on extensive studies of their MICs, pharmacological, clinical outcome, and microbiological parameters [23]. Due to the lack of extensive data, statistical measures were adopted for the process [24]. In this study, based on the clinical outcomes of an effectiveness study of cefquinome against pig streptococcosis, a tentative clinical cutoff was established.

The proposed CO_CL_ should be developed with a therapeutic outcome based on the infection of different MIC isolates. Considering the MIC distribution, the pathogenic clinical isolates of different MICs, which are selected from distribution of sensitive strain, MIC_50_ value, MIC_90_ value, ECOFF, and resistant isolate, were chosen on the basis of pathogenicity experiments performed on mice (data not shown) for the establishment of an experimental model of pig streptococcosis. Then, the therapeutic dose of 2 mg/kg body weight was used for treatment. The pigs in groups *SSZD01* and *SS1496*, which exhibited MICs of 0.015 and 0.06 μg/mL, respectively, had a rapid clinical recovery according to the clinical score. In contrast, in the *SS14130* and *SS2481* groups (1 and 2 μg/mL, respectively), the clinical recovery was unsatisfactory over the whole treatment period, and more failure cases arose from isolates with high MICs. The clinical efficacy of cefquinome against clinical isolates of different MICs was confirmed regarding each group obtaining treatment by a significant reduction in the clinical score. Ultimately, the tentative CO_CL_ was calculated as 0.5 μg/mL.

## 4. Materials and Methods

### 4.1. Determination of the Epidemiologic Cutoff

The MIC of 342 *S. suis* was measured with agar dilution with CLSI guidelines [25]. Strains of *S. suis* were inoculated onto Mueller-Hinton (MH) agar containing sheep blood (5%), with twofold dilutions (0.0075–32 μg/mL) of cefquinome (Dr. Ehrenstorfer, Augsburg, Germany).The plates were incubated at 37 °C in an atmosphere containing 5% CO_2_ for 24 h. *Escherichia coli* (ATCC 25922) was selected as the quality control strain for determination of the MIC. Prior to testing, each isolate was sub-cultured at least three times in trypticase soy agar (TSA) and trypticase soy broth (TSB) containing 5% newborn calf serum (Zhejiang Tian Hang Biotechnology Co., Ltd., Hangzhou, China).

The MIC distribution was constructed and converted into a cumulative log-normal distribution. Then, nonlinear least-squares regression was employed to fit the cumulative log_2_-transformed MIC data to obtain a range of optimum wild-type MIC distributions, which contained the wild-type MIC in the range of 0.1% and 99.9% and to calculate the probability of MIC data falling within the cutoff range. The cutoff value would encompass at least 95% of the wild-type isolates [14]. The ECOFF value could also be calculated by ECOFFinder software, which is a fully automatic Microsoft Excel spreadsheet calculator, following the methodology described by Turnidge. ECOFFinder is freely available on the CLSI website [11].

### 4.2. Pharmacodynamics of Cefquinome against SS0061

#### 4.2.1. MIC and MBC Determination of SS0061 In Vitro and Ex Vivo

*SS0061* strain, for the highly virulent, was selected to research the in vitro and ex vivo antimicrobial activity of cefquinome. For determination of MIC, a 100 μL suspension of *SS0061* at 1 × 10^6^ CFU/mL was added to 100 μL TSB or plasma with cefquinome concentrations from 16 to 0.03 μg/mL followed by the broth dilution method. The plates were incubated at 37 °C for 24 h. An aliquot of 100 μL from each clear tube was sub-cultured on TSA; the plates were incubated at 37 °C overnight, and the colonies were counted to determine the minimum bactericidal concentration (MBC).

#### 4.2.2. In Vitro and Ex Vivo Time-Killing Curve of SS0061

For the in vitro time-killing curves, 5 mL SS0061 at 10^5^ CFU/mL were added to 5 mL TSB with cefquinome concentrations as 0 × MIC, 1 × MIC, 2 × MIC, 4 × MIC, 8 × MIC, 16 × MIC, 32 × MIC and 64 × MIC. Each culture was serially diluted 10-fold with sterile saline, and 100 μL of each dilution from different time points (0, 1, 2, 4, 6, 8, 12, and 24 h) was spread onto TSA agar plates.

For the ex vivo time-killing curves, *SS0061* was cultured at 37 °C in TSB until logarithmic phase and diluted to 10^5^ CFU/mL. *SS0061* were co-incubated with plasma samples collected from healthy and infected pigs at different time points after I.M. administration of cefquinome (2 mg/kg b.w.) (Shanghai Tongren Pharmaceutical Co., Ltd., Shanghai, China). Each culture was serially diluted 10-fold with sterile saline, and 100 μL of each dilution from different time points (0, 3, 6, 9, 12, and 24 h) was spread onto TSA agar plates. Then, the bacterial count (CFU/mL) was determined after incubation for 24 h at 37 °C with 5% CO_2_. The limit of detection was 10 CFU/mL.

#### 4.2.3. Determination of the MPC of Cefquinome against SS0061

An aliquot of 100 μL of the 10^10^ CFU/mL bacterial suspension was cultured on TSA plates containing various concentrations of cefquinome (0 × MIC, 1 × MIC, 2 × MIC, 4 × MIC, 8 × MIC, 16 × MIC, 32 × MIC, and 64 × MIC), incubated for 72 h, and colonies were counted every 24 h. All mutant prevention concentration (MPC) determinations were performed in duplicate. The MPC was defined as the lowest cefquinome concentration with no visible bacterial growth on agar plates after incubation for 72 h.

#### 4.2.4. In Vitro PAE

The PAE of cefquinome is determined as described. *SS0061* was incubated with 1, 2 and 4 MIC of cefquinome. After incubating for 1 or 2 h, the drug was removed by dilution 1000 times with fresh medium. The viable counts of *S. suis* were determined at 1, 2, 4, 6, 8, 10 and 12 h. The PAE was calculated as follows: PAE = T-C, where T and C represent the periods required for viable counts of bacteria to increase by 1-log_10_ CFU in the drug removal phase for the treatment and the untreated control groups, respectively.

### 4.3. Pharmacokinetics of Cefquinome in the Plasma of Pigs

#### 4.3.1. Animals and Dosing

Sixteen pigs, with an average weight of 15 ± 2 kg, were randomly segmented into groups A and B, with 8 pigs in each group. Each pig in group B was inoculated with 3 mL of 1 × 10^8^ CFU/mL *SS0061* by intranasal infection to establish the infected model. Animals in group A were inoculated with an equal volume of TSB broth. When streptococcus’s symptoms such as high temperature, loss of appetite, fatigue, increased breathing rate, coughing, corneal flushing, joint swelling, and central nervous system signs were observed in group B, all the animals were administered cefquinome sulfate at a dose of 2 mg/kg via posterior auricular muscle injection. All healthy animals were returned to the farm.

#### 4.3.2. Sample Collection

Blood samples (5 mL) from each pig in the healthy and infected groups were collected at 0, 0.08, 0.17, 0.25, 0.5, 0.75, 1, 1.5, 2, 3, 4, 6, 8, 10, 12, 24, 36, and 48 h after injection administration from the jugular vein, and transferred into heparinized tubes. Plasma samples were centrifuged at 3500 rpm/min for 10 min. All samples were stored at −20 °C prior to the analysis.

#### 4.3.3. Sample Analysis

The quantitative analysis of cefquinome in plasma and PELF was performed by HPLC-UV. A ZORBAX SB-Aq reverse-phase column (250 mm × 4.6 mm, i.d. 5 μm, Agilent) was used to execute HPLC at a detection wavelength of 265 nm at 30 °C. The mobile phase consisted of 0.05% phosphoric acid (phase A) and acetonitrile (phase B) at a flow rate of 1 mL/min with a gradient elution condition.

Plasma (0.5 mL) was deproteinized with 1 mL of acetonitrile. After centrifugation, 1.5 mL of dichloromethane was added to the supernatant to carry out the back extraction. The top layer was transferred into an autosampler vial and injected into HPLC system after vortex mixing and centrifugation. The standard curves of cefquinome were linear from 0.08 to 10 μg/mL in plasma (R^2^ = 0.9966). The limit of determination (LOD) was 0.03 and the limit of quantification (LOQ) was 0.08 μg/mL in plasma. The recovery of cefquinome in plasma ranged from 79.57% to 93.20%.

### 4.4. Pharmacokinetic/Pharmacodynamic Integration Model

The PK/PD integration model was calculated with the parameters representing the bacteriological outcome. Then, the inhibitory sigmoid *I_max_* model was used to simulate the values of PK/PD parameters by WinNonlin software version 5.2.1. The model equation is described as follows:

$$E=E_{0}-\frac{I_{max}\times C^{N}}{C^{N}+EC_{50}^{N}}$$

where *E* is the antibacterial effect measured as the change in the bacterial count (log_10_ CFU/mL) in the plasma sample after 24 h of incubation compared to the initial value, *E*_0_ is the maximum antibacterial effect determined as the difference in the log_10_ CFU/mL value of the sample incubated between 0 h and 24 h, *I_max_* is the amplitude of maximal effect, *EC*_50_ is the value of PK/PD parameter which can produce 50% of the maximum antibacterial effect, *C* is the PK/PD parameters in the effect compartment, and *N* is the Hill coefficient, which describes the steepness of the PK/PD parameter-effect curve.

Three levels of the antibacterial effect were quantified from the sigmoid Emax model by PK/PD parameters required for static action (no change in bacterial counts after 24 h incubation, *E* = 0), 1-log_10_ killing effect (a 90% reduction in bacterial count, *E* = −1), 2-log_10_ killing effect (a 99% reduction in the bacterial count, *E* = −2) and 3-log_10_ killing effect (a 99.9% reduction in the bacterial count, *E* = −3) in each of the samples.

### 4.5. Determination of CO_PD_ by Monte Carlo Simulation

In order to obtain the probability target attainment, PK parameters and PK/PD targets are selected for simulation. PK/PD targets corresponding to different antibacterial effect were selected to obtain the probability of target attainment (PTA). CO_PD_ was defined as the maximal MIC value at which the corresponding PTA was ≥90% [26].

### 4.6. Dose Estimations

PK/PD parameters corresponding to an *E* value in the plasma were used to deduce an optimal dose regimen. The potential optimal dosage could be calculated as the equation:

$$Dose=\frac{{\left(\frac{AUC}{MIC}\right)}_{ex}\times MIC}{fu}\times CL/F$$

where *MIC* is 0.25 μg/mL (MIC_90_) of cefquinome against *S. suis*, (*AUC/MIC*)*_ex_* is the target end point for optimal efficacy, *CL/F* is the clearance per fraction absorbed, and unbound fraction (*fu)* is 0.812 [27].

### 4.7. Clinical Effectiveness of Cefquinome on Pigs Infected with Different S. suis Isolates

#### 4.7.1. Animal Groups and Establishment of Infected Model

Thirty-six piglets, with an average weight of 20 ± 4 kg, were randomly segmented into 6 groups, one of which was a blank control group with 6 pigs, and the other five with 6 pigs in each group were infected with five different strong pathogenic *S. suis* strains, In this study, these strains corresponded to the strains of *SSZD01*, *SS1496*, *SS0061*, *SS14130*, *SS2481*, and their MIC value was 0.015 μg/mL, 0.06 μg/mL, 0.25 μg/mL, 1 μg/mL, 2 μg/mL, respectively. The infected model was established by inoculating with 3 mL of 1 × 10^8^ CFU/mL *SS0061* via nasal inoculation. The infected pigs in each group were administered with cefquinome sulfate injection when *streptococcosis* symptoms were observed. Piglets were observed daily for rectal temperature, respiratory state and behavior and depression. The main observing targets were scored following criteria described in the FDA guidance with some improvements.

#### 4.7.2. Statistical Analysis of Clinical Outcome and Clinical Cutoff

CLSI and EUCAST have not yet published the unified procedure for establishing CO_CL_. Three analysis methods could be used to analyze the clinical efficacy data, including Classification and Regression Tree analysis (CART), WindoW approach, and nonlinear regression analysis. For CART, the software of Salford Predictive Modeler version 8.2 was used to predict the likelihood of success or failure in cefquinome-treated piglets with *S. suis* infection. WindoW approach is a proposal for CLSI/VAST discussion [28]. This is accomplished using two separate algorithms (MaxDiff and CAR), simultaneously applied to define a Window of CO_CL_. The range of CO_CL_ values could be determined by both methods. Then the relationship between log_2_ MIC for 5 isolates and cure rate derived from clinical effectiveness studies was simulated by nonlinear regression analysis. CO_CL_ was defined as the maximum MIC value with a cure rate of 90% under the same dosing regimen.

### 4.8. Development of Susceptibility Breakpoints

The final susceptibility breakpoint of cefquinome against *S. suis* was established based on the (SBPT) decision tree with the TECOFF and CO_PD_ and CO_CL_ values.

## 5. Conclusions

This was the first study on susceptibility breakpoint and the optimal dosage regimen of cefquinome against *S. suis* in pigs. The TECOFF was determined as 1 μg/mL, the CO_PD_ was calculated of 0.06 μg/mL and the CO_CL_ was determined as 0.5 μg/mL. Based on the (SBPT) decision tree, the susceptibility breakpoint was set as 1 μg/mL. The optimized dose was determined as 3.08 mg/kg for the clinical treatment. This study offers evidence for monitoring resistance emergence and guidance for clinical treatment.

## Figures and Tables

**Table 1:** The upper limit and probability of MIC distribution.

Subset Fitted	MIC (μg/mL)	True	Estimate	Diff.	Probabilities
97.5% Subset ECOFF	0.25	311	332	21	90.9%
99% Subset ECOFF	0.5	328	340	12	95.9%
99.9% Subset ECOFF	1	337	340	3	98.5%

Note: True is the actual cumulative numbers of strains; Estimate is the simulative cumulative numbers of strains; Diff is the difference between True and Estimate.

**Table 2:** Post-antibiotic effect (PAE) after 1 and 2 h on *SS0061*.

Concentrations	PAE (h)	
	Expose 1 h	Expose 2 h
1 × MIC	0.12	0.68
2 × MIC	0.62	0.74
4 × MIC	1.04	1.24

**Table 3:** Pharmacokinetic parameters of cefquinome in plasma at a dose of 2 mg/kg by intramuscular injection of the pigs (mean ± SD).

Parameters	Units	Plasma	
		Healthy (n = 8)	Infected (n = 8)
α	1/h	4.73 ± 0.79	4.54 ± 0.94
β	1/h	0.31 ± 0.17	0.34 ± 0.16
T_1/2α_	h	0.15 ± 0.03	0.16 ± 0.03
T_1/2β_	h	2.24 ± 0.23	2.33 ± 0. 21
T_max_	h	0.45 ± 0.02	0.42 ± 0.03
AUC	h·μg/mL	9.77 ± 0.63	9.86 ± 0.78
C_max_	μg/mL	3.93 ± 0.11	3.92 ± 0.13
CL/F	mL/kg/h	204.51 ± 19.50	202.77 ± 17.03
Vd/F	L/kg	0.11 ± 0.06	0.10 ± 0.02

Note: AUC: the area under the concentration-time curve; C_max_: maximal drug concentration; T_max_: time to reach C_max_; T_1/2α_: distribution phase half-life; T_1/2β_: elimination half-life; α: distribution phase rate constant; β: elimination phase rate constant; CL/F: clearance per fraction absorbed; V_d_/F: volume of distribution per fraction absorbed.

**Table 4:** The PK/PD modeling of cefquinome in plasma at a dose of 2 mg/kg by intramuscular injection of the pigs.

Parameters	Units	Healthy	Infected
I_max_	Log CFU/mL	6.53 ± 0.13	6.00 ± 0.25
E_0_	Log CFU/mL	2.89 ± 0.19	2.92 ± 0.47
EC_50_	h	79.14 ± 2.44	46.38 ± 4.48
N	-	7.54 ± 0.61	24.81 ± 10.12
Static effect	h	76.73 ± 1.84	46.00 ± 4.53
1-log_10_ killing	h	83.32 ± 1.84	47.59 ± 3.95
2-log_10_ killing	h	91.54 ± 1.96	49.66 ± 3.34
3-log_10_ killing	h	106.44 ± 3.28	-

**Table 5:** The efficacy of cefquinome for the treatment of pig *streptococcicosis* caused by different MIC isolates.

Groups	MIC (μg/mL)	Mortality Rate (%)	Cure Rate (%)	AUC_succ_	AUC_total_	CAR	%Success ≤ MIC	MaxDiff
*SSZD01*	0.015	0	100	0.05	0.05	1.00	100.00	25.00
*SS1496*	0.06	0	100	0.32	0.32	1.00	100.00	33.30
*SS0061*	0.25	17	83	1.36	1.46	0.93	83.33	25.03
*SS14130*	1	33	67	4.74	5.96	0.80	66.67	16.67
*SS2481*	2	50	50	8.24	11.96	0.69	50.00	0.00

Note: AUC_Succ_: AUC based upon the number of therapeutic successes; AUC_Total_, AUC estimated based upon the total observations; CAR: cumulative AUC ratio; MaxDiff: the method of maximum difference.

## Data Availability

Data is contained within the article.

## Abbreviations

AUC: Are under concentration; CART: Classification And Regression Trees; CLSI: Clinical laboratory standard institution; CO_PD_: Pharmacodynamics cutoff; CO_CL_: Clinical cutoff; CFU: Colony-forming unit; ECOFF: Wild cutoff; fu: unbound faction of antibiotic; HPLC: High Performance Liquid Chromatography; MBC: Minimum bactericidal concentration; MIC: minimum inhibitory concentration; MPC: mutation preventive concentration; MSW: mutant selection windows; PAE: Post-antibiotic effect; PK: Pharmacokinetic; PD: Pharmacodynamics; PTA: Probability of target attainment; SBPT: Susceptibility breakpoints; *S. suis*: Streptococcus suis; TECOFF: Terminal wild cutoff; T > MIC: Time above minimum inhibitory concentration.
